# Evaluation of Intraosseous Excretory Urography Using Iodixanol for Renal Imaging in Budgerigars (*Melopsittacus undulatus*)

**DOI:** 10.1002/vms3.70750

**Published:** 2025-12-23

**Authors:** Hamideh Zeinali, Ali Mirshahi, Peyman Nakhaee, Mohammad Azizzadeh

**Affiliations:** ^1^ Private Veterinarian Mashhad Iran; ^2^ Department of Clinical Sciences Faculty of Veterinary Medicine, Ferdowsi University of Mashhad Mashhad Iran; ^3^ Private Avian Specialist in Veterinary Medicine Mashhad Iran

**Keywords:** budgerigar, contrast media, excretory urography, intraosseous, kidney

## Abstract

**Background:**

Renal diseases in budgerigars (*Melopsittacus undulatus*) are frequently encountered in clinical practice and represent a significant concern for avian veterinarians. Among the available diagnostic methods, each with its advantages and limitations, excretory urography is considered a valuable tool for confirming and accurately localising renal and urinary tract system. However, due to the small body size of birds, their thin‐walled vessels, and the risk of vascular collapse under various conditions, intravenous (IV) access is often difficult or unfeasible.

**Objectives:**

This study aimed to evaluate the feasibility and safety of intraosseous injection (IO) of iodixanol, a nonionic iodinated contrast medium (CM), via the proximal tibiotarsus for producing diagnostic excretory urograms in budgerigars.

**Methods:**

Twenty clinically healthy adult budgerigars were selected and serial radiographs were taken at multiple time points: 0, 1, 2, 3, 4, 5, 10, 15, 20, 30, 60 min and 24 h after IO injection of iodixanol.

**Results:**

CM distribution in the kidneys, ureters and cloaca was observed immediately post‐injection and at the time of maximum renal opacity. Statistical analysis revealed no significant differences in the length or height of the cranial part of kidney among the three measured time points.

**Conclusion:**

This study demonstrates that excretory urography using intraosseous administration of iodixanol into the proximal tibiotarsus is a safe, practical, and well‐tolerated technique, with no side effects, for accurate diagnosis of renal and urinary tract disorders in budgerigars.

## Graphical abstract

This study demonstrates that intraosseous injection of iodixanol into the proximal tibiotarsal bone provides a reliable, safe, and practical method for diagnostic excretory urography in budgerigars. The technique offers clear visualisation of renal structures and can be an alternative when intravenous access is limited in avian patients.

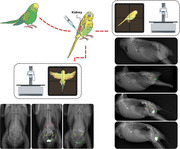

## 1 Introduction

The budgerigar (*Melopsittacus undulatus*) is one of the most widely kept ornamental birds, and consequently, the number of clinical cases presented to veterinary clinics has increased. This highlights the need for improved diagnostic tools that can support disease prevention, accurate treatment, and overall enhancement of health and welfare in this species (Simova‐Curd et al. 2006; Velayati et al. 2015). Diagnosis of renal disorders in birds is particularly challenging, as pathognomonic clinical signs are uncommon and renal disease frequently occurs alongside other conditions (Lierz 2003). Among common avian disorders, renal and urinary tract diseases are frequently encountered in psittacines, especially budgerigar, with different causes ranging from toxins and infections to nutritional deficiencies and neoplasia (Styles and Phalen 1998). Early and accurate diagnosis of such conditions is therefore essential (Lierz 2003; Pollock 2006). A wide range of diagnostic techniques and approaches including conventional radiography, biochemistry analysis of blood plasma (uric acid, sodium, potassium, and phosphorus), urinalysis (pH, color, osmolality, protein, glucose and enzymes), ultrasonography, computed tomography (CT), magnetic resonance imaging (MRI), endoscopy and kidney biopsies are currently implemented for detection of kidney and urinary tract disturbances (Krautwald‐Junghanns and Konicek 2020; Lierz 2003; Pollock 2006). However, each method has limitations. For example, radiographic interpretation may be hindered by the superimposition of kidney structures with active gonads, endoscopy and biopsy are invasive, and collecting blood samples for biochemical analysis poses risks in small pet birds (Burgos‐Rodríguez 2010; Krautwald‐Junghanns and Konicek 2020; Suedmeyer and Bermudez 1996; Tripathi et al. 2011). Avian kidneys and urinary structures are often difficult to distinguish on plain radiographs due to cysts, tumours, or overlap with adjacent tissues of similar radiodensity. In such cases, excretory urography can improve visualisation, as it relies on renal‐selective concentration and excretion of contrast medium (Heuter 2005; Moulvi et al. 2013). Iodine‐based contrast agents, both ionic and nonionic, are available for this purpose. Modern nonionic agents are generally preferred in birds due to their reduced side effects (Krautwald‐Junghanns et al. 2008). Iodixanol, an iso‐osmolal, nonionic, dimeric, hydrophilic contrast medium is particularly suitable because of its short transit time and minimal adverse effects (Martel et al. 2018). Excretory urography thus serves as a valuable tool for both morphological and functional evaluation of the avian urinary tract and for distinguishing kidneys from adjacent structures, radiographically (Krautwald‐Junghanns et al. 2008). Various routes of CM administration have been described, including intravenous (IV), intraosseous (IO), intramuscular (IM), intracardiac (IC) and subcutaneous (SC). However, in small avian species, IV and SC access are often limited by fragile, small‐calibre vessels, risk of vascular collapse, soft tissue trauma, or oedema, making these routes impractical (Jania et al. 2022; Porzio et al. 2001). Maintaining IV access is further complicated by the limited subcutaneous tissue surrounding peripheral vasculature. For these reasons, IO catheterisation is frequently used as a practical alternative in birds (Jania et al. 2022). The objective of the present study was to evaluate the morphology and function of kidneys and urinary tract in budgerigars using IO injection of iodixanol, aiming to establish a safe and reliable method for diagnostic excretory urography. In other words, this study aims to evaluate the feasibility and safety of IO administration of iodixanol for diagnostic excretory urography in budgerigars.

## 2 Materials and Methods

### 2.1 Sample Collection

This study was approved by the ethics committee of the Faculty of Veterinary Medicine of the Ferdowsi University of Mashhad, Iran (IR.UM.REC.1400.374). Twenty clinically healthy adult budgerigars (*Melopsittacus undulatus*) (10 males and 10 females), with no clinical evidence of renal or urinary tract disorders, were obtained from a private avian breeder and acclimated for 2 weeks prior to experiment. During this period, food and water intake, cage hygiene, husbandry conditions, droppings, feather appearance, body condition, and cardiorespiratory parameters were monitored daily. The apparent health status of all birds was confirmed through physical examination by an avian specialist. In addition, complete blood chemistry and urinalysis were performed to further verify the clinical health of all experimental birds.

### 2.2 Housing and Husbandry Conditions

All birds were maintained in a controlled experimental room throughout the study period. Room temperature and humidity were monitored daily using a digital thermoshygrometer (TROTEC, Germany). A 12‐h light/dark cycle was provided with artificial lighting controlled by a timer (TFA Dostmann, Germany). Birds were housed in pairs in wire cages (60 × 60 × 60 cm), each equipped with wood perches and feeding bowls. Food and water were provided ad libitum. The diet consisted of a daily combination of commercial seed mixture (Versele‐Laga Premium Prestige, Belgium) and a pelleted formulation (FruitBlend Flavor with Natural Flavors, USA). During the 2 weeks of husbandry and prior to the beginning of the experimental procedures, each bird was weighed using a small digital scale (Tanita, China) following gentle restraint with a towel. Body weights ranged from 26.70 to 43.60 g (mean ± SD: 33.71 ± 4.22 g).

### 2.3 Urography Procedure

**FIGURE 1:**
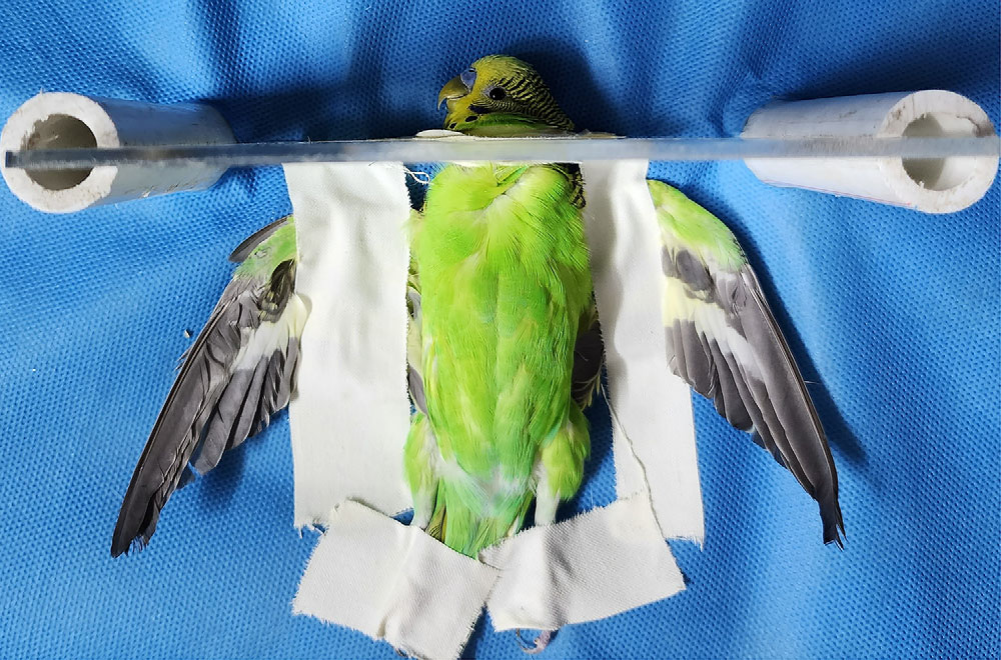
Restrain of each budgerigar during the experiment using handmade positioning equipment constructed of radiolucent Plexiglas boards.

**FIGURE 2:**
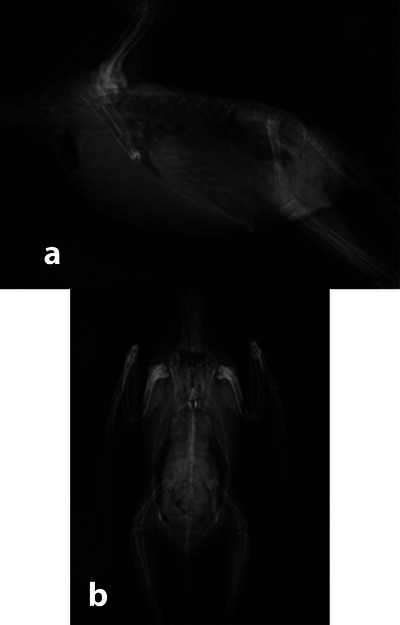
Two plain right lateral (RL) (a) and ventrodorsal (VD) (b) radiographic views were provided for assessment of celomic cavity of each budgerigar before injection of contrast media.

As illustrated in Figure 1, each bird was manually restrained using a handmade positioning device constructed from radiolucent Plexiglas boards. Ventrodorsal (VD) radiographs were obtained with the birds in dorsal recumbency, and then affixing the legs and wings extended laterally to another larger Plexiglas board. A smaller mobile portion of the device supported the cervical region. Prior to CM administration, two control radiographs—right lateral (RL) and VD—were obtained to evaluate the celomic cavity (Figures 2a, b).

**FIGURE 3:**
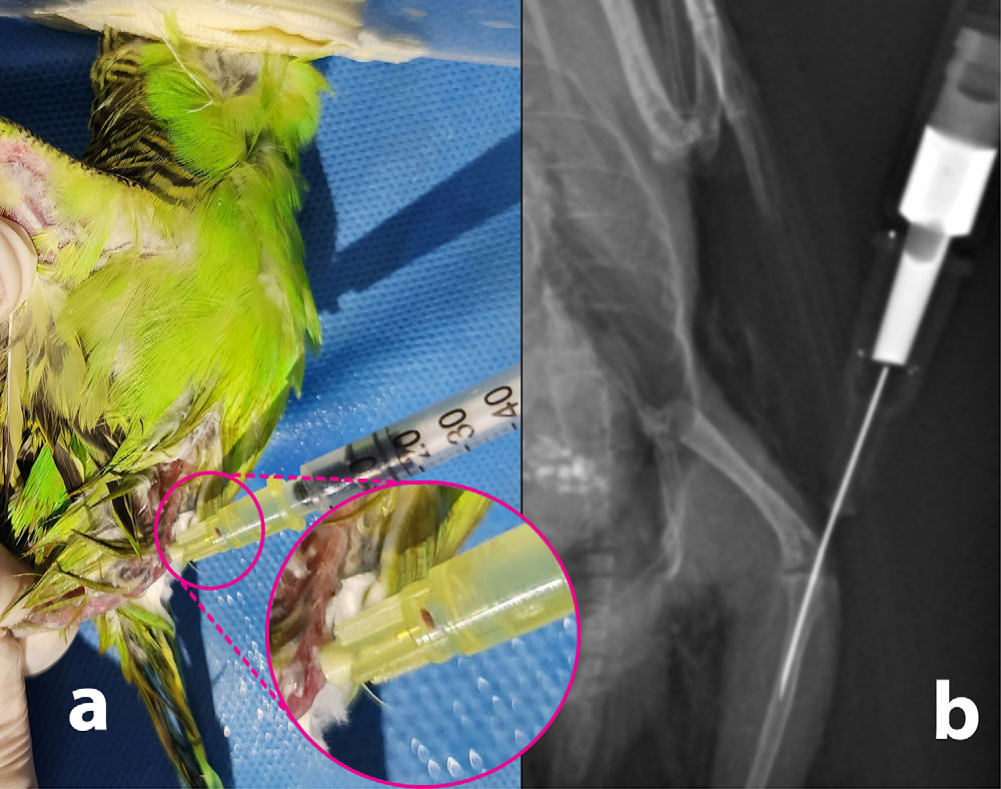
Ventrodorsal radiographic view of intraosseous injection of iodixanol into the medullary cavity of tibiotarsal bone (b) and subsequent presence of blood in the needle hub indicating accurate method and location of intraosseous injection (a).

All birds were sedated with intranasal administration of 13.2 mg/kg of midazolam (Zodalem 5 mg/mL, Caspian Tamin Pharmaceutical Co, Iran) (Sadegh 2013). Standard aseptic preparation was performed prior to the IO injection. Excretory urography was conducted using iodixanol (Visipaque 320 mgI/mL, GE Healthcare, Norway), a nonionic CM. The agent was injected into the proximal tibiotarsal bone via the IO rout using a 1‐mL syringe and a 31‐gauge catheter at a dose of 2 mL/kg (Figures 3a, b).

Following CM administration, sequential RL radiographs were obtained at the following time points: immediately after injection (first obtainable image, approximately 10–20 s), 1, 2, 3, 4, 5, 10, 15, 20, 30, and 60 min, as well as 24 h post‐injection. Complete flushing was performed after each CM administration. Radiographs were taken in the RL and VD projections using a commercial radiographic unit (Siemens, Multix top, Erlangen, Germany) and a digital radiography flat panel detector (XRpad2, Varex, Walluf, Germany). RL projections were considered superior for excretory urography due to reduced superimposition of visceral organs over the kidneys. Radiographic parameters included a focal‐film distance of 110 cm, 55 kVp, and 2.8 mAs. All birds were monitored for 24 h post‐procedure to detect any abnormalities. The following parameters were evaluated: (1) measurement of length and height of cranial part of the kidneys pre‐injection of CM, at time of maximum renal opacity and at 24 h post‐injection of CM; (2) time from CM administration to maximum renal opacity; (3) time from CM administration to complete renal clearance; and (4) timing of visualisation of celomic cavity structures including ureters and cloaca.

### 2.4 Statistical Analysis

Descriptive statistics (mean, standard deviation [SD], minimum, maximum, first quartile, and third quartile) were calculated for all parameters. Data were analysed using SPSS software (version 25.0, IBM Corp., Armonk, NY, USA). A repeated‐measures ANOVA was performed to assess differences across time points. Statistical significance was set at *p* < 0.05.

## 3 Results

**FIGURE 4:**
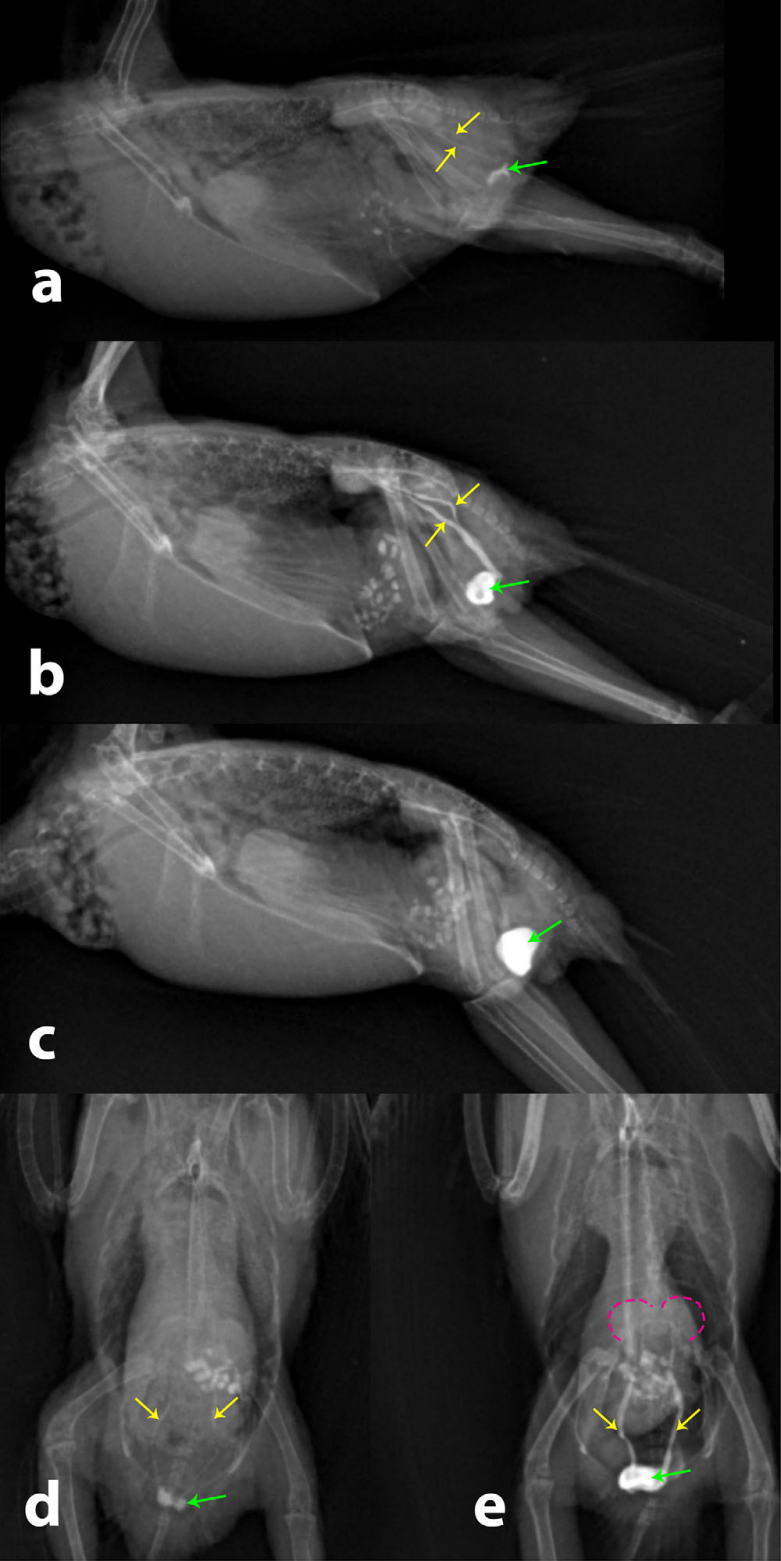
Right lateral and ventrodorsal radiographic views of immediate increased opacity of kidneys, ureter and cloaca at zero‐time post‐injection (yellow arrows: ureters; green arrows: cloaca) (a, d); presence of contrast media in kidney, ureter and cloaca at right lateral and ventrodorsal radiographic views at the time of maximum opacity of kidneys (yellow arrows: ureters; green arrows: cloaca; dashes: kidneys) (b, e); time of clearance of CM from kidneys and ureters at RL radiographic view (green arrow: cloaca) (c).

**FIGURE 5:**
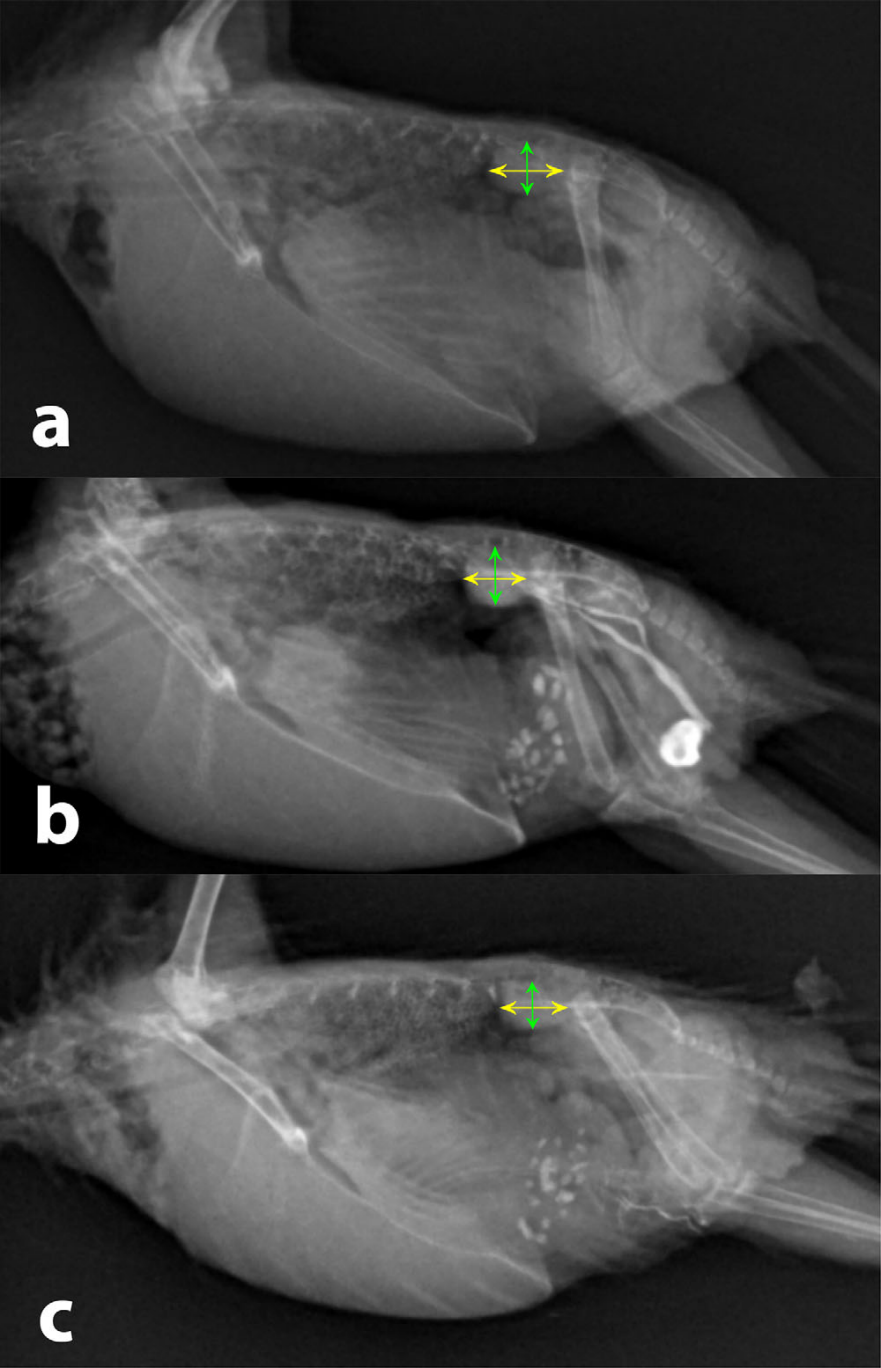
Right lateral radiographic views of length (yellow arrows) and height (green arrows) of cranial part of the kidneys pre‐injection of CM (a), at the time of maximum opacity of kidneys (b) and 24 h post‐injection of CM (c).

All budgerigars remained clinically normal, with no iodixanol‐related adverse effects observed during one month of follow‐up. Intraosseous administration of CM resulted in an immediate increase in the radiographic opacity of the kidneys, ureters, and cloaca (Figures 4a, d). The presence of CM in these organs and structure was confirmed in both VD and RL projections at time zero post‐injection and at the point of maximum renal opacity (Figures 4b, e). Clearance of CM from the kidneys and ureters was evident on RL projections, at which stage CM was visible only in the cloaca (Figure 4c). Furthermore, RL radiographs demonstrating the renal length and height were obtained at three time points: pre‐injection, maximum renal opacity, and 24 h post‐injection (Figure 5a–c). The mean, standard deviation (SD), confidence interval (CI), minimum and maximum values of the length of cranial renal part at these three time points are presented in Table 1. Statistical analysis revealed no significant differences in length of cranial part of kidney across the three measurements (*p* > 0.05). Comparable findings were obtained for height of cranial part of kidney, with no statistically significant differences observed among the three time points (Table 2, *p* > 0.05).

**TABLE 1:** Summary of data obtained from measured length of cranial part of kidneys at three different times and no significant difference was observed (*p >* 0.05).

					95% Confidence interval	
Length (mm)	Number	Mean	Min–Max	SD	Lower limit	Upper limit
Pre‐injection of CM	20	5.86	5–6.77	0.49	5.63	6.09
Time of maximum opacity of kidneys	20	5.91	4.27–7.07	0.66	5.60	6.23
24 h post‐injection of CM	20	5.72	4.67–6.97	0.58	5.45	5.99

Abbreviations: CM, contrast media; Max, maximum; Min, minimum; SD, standard deviation.

**TABLE 2:** Summary of data related to the height of cranial part of kidneys at three different times and no significant difference was observed (*p >* 0.05).

					95% Confidence interval	
Height (mm)	Number	Mean	Min–Max	SD	Lower limit	Upper limit
Pre‐injection of CM	20	3.94	5.33–2.80	0.53	3.69	4.19
Time of maximum opacity of kidneys	20	4.22	5.67–3.23	0.64	3.92	4.53
24 h post‐injection of CM	20	4.20	6.47–3.10	0.68	3.88	4.52

Abbreviations: CM, contrast media; Max, maximum; Min, minimum; SD, standard deviation.

The time to maximum renal opacity and complete clearance of CM from kidneys was also evaluated, and the results are summarised in Table 3. Immediately following iodixanol injection, increased renal opacity in both VD and RL projections was observed in 19 (95%) of the budgerigars, whereas one bird (5%) demonstrated CM uptake at 1 min post‐injection. Ureteral opacification was visible immediately in 18 (90%) birds, while 2 (10%) showed ureteral CM presence at 1 min. Similarly, iodixanol was detected in the cloaca immediately post‐injection in 17 (85%) birds, while 3 (15%) exhibited CM presence in the cloaca at 1 min post‐injection.

**TABLE 3:** Results related to the time of maximum opacity and clearance of kidneys from CM.

Time (min)	Number	Median	First quarter	Third quarter	Min–Max
Time of maximum opacity of kidneys	20	3.00	2.00	3.00	5.00–1.00
Time of clearance of kidneys	20	60.00	30.00	60.00	60.00–20.00

Abbreviations: CM, contrast media; Max, maximum; Min, minimum.

## 4 Discussion

Plain radiography alone is often insufficient for accurate evaluation of the avian kidneys due to superimposition with other visceral organs and active gonads, as well as the difficulty in distinguishing them from renal neoplasms. When CT imaging is not available, administration of CM can provide a more detailed and reliable visualisation of the kidneys and urinary tract. In the present study, IO excretory urography was successfully performed in budgerigars, demonstrating its value as a practical and well‐tolerated technique and without adverse effect for accurate diagnosis of renal and urinary tract disorders in small pet birds. Our findings suggest that the IO rout is the most suitable method of CM administration in small avian species, particularly because thin‐wall blood vessels, difficulties in catheterisation, and subcutaneous oedema arising from various diseases conditions often preclude IV access.

However, a limitation of the IO approach was observed in birds with metabolic bone disease, where CM leakage into surrounding muscles and subcutaneous tissue occurred following needle insertion. Various CM agents have been utilised in human and veterinary excretory urography (Burgos‐Rodríguez 2010; Cochran et al. 1980; Gavant et al. 1992; Heuter 2005; Krautwald‐Junghanns et al. 2008; Moulvi et al. 2013). Iodixanol, in particular, has demonstrated minimal metabolism and excellent renal safety in humans (McCullough 2006; Reed et al. 2009), and this safety profile has also been confirmed in recent veterinary investigations as well as in the current study (Heuter 2005; Krautwald‐Junghanns and Konicek 2020; Krautwald‐Junghanns et al. 2008).

A recent study reported that although IO catheter placement may be technically more challenging, manipulation during imaging is easier, catheter removal does not lead to complications, and adverse effects are minimal (Jania et al. 2022). These findings are consistent with our results, as no lesions or side effects were observed up to 24 h following CM injection. Consistent with our findings, Zangisheh et al. (2025) demonstrated that IO excretory urography is a safe contrast imaging modality and can be effectively applied to assess the urinary system in broiler chickens, particularly for evaluating changes in kidney size and function.

Moreover, Jania et al. (2022) demonstrated that intraosseous (IO) administration of iodinated contrast medium (CM) provided superior enhancement compared with intravenous (IV) administration in CT imaging of Hispaniolan Amazon parrots. This finding further supports the diagnostic value of IO contrast delivery in psittacine species and is consistent with our results, which showed clear representation of CM in the kidneys, ureters, and cloaca following IO injection of iodixanol. Similar to the current study, Jania et al. (2022) introduced IO CM injection as a safe route of administration for excretory urography and diagnostic imaging in birds.

In related studies (Porzio et al. 2001; Sağlam et al. 2004), the authors concluded that urographic imaging via IO CM administration is safe and feasible in rabbits. Furthermore, this approach has been suggested as an effective and reliable alternative to IV urography in paediatric and adult human patients, particularly in emergencies when IO access is the only available vascular route. These findings are in accordance with our study, which also confirmed that IO injection of iodixanol is a safe, complication‐free method for evaluating renal and urinary structures in budgerigars.

In the present study, we additionally demonstrated the presence of CM in the cloaca and observed normalisation of renal opacity to pre‐injection levels, which we interpret as an indicator of renal clearance. Similar findings were reported by Krautwald‐Junghanns and Konicek (2020), who noted that the renal portal system in birds facilitates rapid CM excretion. During urographic examinations, the kidneys and ureters typically become visible 30–60 s post‐injection, which is in agreement with our observations.

In earlier human medicine, excretory urography with IV or subcutaneous (SC) injection of iohexol was widely used for diagnosing urinary tract infections and other paediatric urological disorders, often in combination with renal ultrasound (Drachman et al. 1984; Leonidas et al. 1985; Pollack and Banner 1985; Wyatt 1941). Although modern diagnostic techniques have largely replaced this method, researchers and practitioners of that time emphasised its safety and clinical utility.

Looking forward, further efforts are needed to advance diagnostic tools for avian diseases, particularly renal disorders in small psittacine species.

## 5 Conclusion

This study introduces and validates IO excretory urography with iodixanol as a safe, practical, effective, and without adverse effect method for diagnosis renal and urinary tract system disorders and diseases in budgerigars. Compared with other diagnostic techniques—which may be cumbersome, invasive, or technically unfeasible in small‐sized birds—IO excretory urography offers reliable visualisation of the renal system without adverse effects. While IV or SC routes are often limited by fragile vasculature, risk of vascular collapse, or soft tissue complications, the IO route provides a viable alternative.

When performed correctly, excretory urography can yield critical diagnostic information that may not be obtained through other method. Although biochemical assays, urinalysis, ultrasonography, CT, and MRI remain valuable tools, excretory urography should not be overlooked. Despite the slightly greater technical challenge of IO injection, this approach should be considered a preferred option for small psittacines, with the route of administration ultimately determined by clinician expertise, bird size, and individual conformation. On the other hand, future investigations could focus on evaluating and comparing the feasibility, safety, and potential advantages of intravenous and intracelomic administration of CM, thereby providing deeper insights into their relative applicability in both experimental and clinical settings. Moreover, this technique may serve as a valuable tool in follow‐up investigations of clinical cases, particularly for monitoring disease progression, assessing therapeutic efficacy, or conducting experimental studies involving pathological conditions of the kidneys

## Author Contributions

**Ali Mirshahi**: Conceptualisation, data curation, investigation, validation, visualisation, writing – original draft, methodology, supervision, funding acquisition, project administration, resources, writing – review & editing. **Mohammad Azizzadeh**: Formal analysis, software, investigation, validation, visualisation, writing – review & editing, methodology, conceptualisation. **Peyman Nakhaee**: Investigation, writing – original draft preparation, conceptualisation, investigation, writing – review and editing. **Hamideh Zeinali**: Data curation, software.

## Funding

This research was supported by the Research Council of Ferdowsi University of Mashhad, Mashhad, Iran.

## Ethics Statement

This study was approved by the ethics committee of the Faculty of Veterinary Medicine of the Ferdowsi University in Mashhad, Iran (IR.UM.REC.1400.374). Ethical considerations were paramount throughout the study. Participants provided informed consent, ensuring they understood the study's purpose and their rights. Confidentiality measures were strictly implemented to protect participants’ privacy, and efforts were made to minimise any potential harm or discomfort.

## Conflicts of Interest

The authors declare no conflicts of interest.

## Data Availability

The data that support the findings of this study are available from the corresponding author upon reasonable request.
